# Predicting Abdominal Aortic Aneurysm Progression: The Role of 18F-Fluorodeoxyglucose Aortic Wall Uptake and Flow Dynamics

**DOI:** 10.3390/life16081308

**Published:** 2026-08-10

**Authors:** Marta Ferrer-Cornet, Marvin Garcia-Reyes, Mireia Bragulat-Arévalo, Andrea Guala, Juan Garrido-Oliver, Alba Catala-Santarrufina, Pere Lopez-Gutierrez, Ruperto Oliveró-Soldevila, Gisela Teixidó-Turà, Ignacio Ferreira-Gonzalez, Jose Rodriguez-Palomares, Sergi Bellmunt-Montoya, Lydia Dux-Santoy

**Affiliations:** 1 Vall d’Hebron Institut de Recerca (VHIR), 08035 Barcelona, Spainmireia.bragulat@vhir.org (M.B.-A.); juan.garrido@vhir.org (J.G.-O.); alba.catala@vhir.org (A.C.-S.); pere.lopez@vhir.org (P.L.-G.); gisela.teixido@vallhebron.cat (G.T.-T.); ignacio.ferreira@vallhebron.cat (I.F.-G.); sergi.bellmunt@vallhebron.cat (S.B.-M.); lydia.duxsantoy@vhir.org (L.D.-S.); 2 Department of Vascular Surgery, Hospital Universitari Vall d’Hebron, 08035 Barcelona, Spain; marvinernesto.garcia@vallhebron.cat; 3 Department of Medicine, Universitat Autònoma de Barcelona, 08193 Bellaterra, Spain; 4 Centro de Investigación Biomédica en Red de Enfermedades Cardiovasculares (CIBER-CV), Instituto de Salud Carlos III, 28029 Madrid, Spain; 5 Department of Cardiology, Hospital Universitari Vall d’Hebron, 08035 Barcelona, Spain; rupertocarlos.olivero@vallhebron.cat; 6 Centro de Investigación Biomédica en Red de Epidemiología y Salud Pública (CIBERESP), Instituto de Salud Carlos III, 28029 Madrid, Spain

**Keywords:** abdominal aortic aneurysm progression, thrombus, hemodynamics, 18F-fluorodeoxyglucose positron emission tomography, 4D flow cardiovascular magnetic resonance

## Abstract

As abdominal aortic aneurysms (AAA) rupture results in a high mortality rate, early detection and risk stratification are critical for timely elective intervention. Although the maximum diameter is the sole imaging metric used to assess risk, other factors may provide complementary information. This study investigated the role of inflammation and hemodynamics in AAA progression. **Methods**: Patients with AAA underwent hybrid 18F-fluorodeoxyglucose ([18F]FDG) PET/CMR imaging, including a four-dimensional flow sequence and contrast-enhanced magnetic resonance angiography, and were followed to assess AAA diameter growth rate. Abdominal aorta hemodynamics and [18F]FDG PET target-to-background (TBR) were quantified at 28 standardized regions. Correlations between AAA diameter, hemodynamics, PET and growth rate were assessed. **Results**: Thirty-two AAA patients (84.4% men) with a median age of 73 and an interquartile range of [68; 77] years were prospectively recruited. The AAA maximum diameter was 48.1 [44.8; 55.0] mm, and the growth rate was 2.7 [1.3; 4.0] mm/year during a follow-up of 2 [1.1; 3.5] years. The AAA maximum diameter (R = 0.443, *p* = 0.011) and flow vorticity (R = 0.453, *p* = 0.009) showed significant bivariate correlation with the growth rate. When correcting for the aneurysm diameter, the helicity density was significantly correlated with the growth rate (*p* = 0.045), while the AAA thrombus volume was inversely associated with it (*p* = 0.042). Aneurysms with negative helicity density (counterclockwise rotation) grew significantly faster (*p* = 0.042) than those with positive helicity. In multivariable analysis, beyond the AAA diameter, the thrombus volume, helicity density and TBR were inversely related to the growth rate. **Conclusions**: Integrating flow, inflammatory and thrombus information may improve AAA growth rate prediction beyond the maximum aneurysm diameter.

## 1. Introduction

An abdominal aortic aneurysm (AAA) is a pathological focal dilation of the abdominal aorta that can grow and rupture, resulting in a very high mortality rate [1]. Beyond the control of risk factors, such as smoking and hypertension, the treatment options for AAA patients are elective open surgery or endovascular aortic repair, both resulting in relatively low 30-day mortality [2]. However, both interventions are associated with perioperative complications, such as an inflammatory response following surgery [3], as well as long-term complications [4]. Therefore, it is crucial to understand the factors leading to AAA growth so that patients can be stratified according to their risk and elective intervention can be timed to meet individual patients’ needs.

AAA is more prone to rupture with increased aneurysm diameters [5]. In addition to the maximum AAA diameter, other imaging-based features, clinical factors and circulating biomarkers have been associated with AAA progression. Among clinical factors, current smoking [6] and family history [7] are associated with increased growth, while diabetes mellitus decreases growth [6,8]. Several clinically used circulating biomarkers, including D-dimer [9], total cholesterol and apolipoprotein-B [10], as well as experimental biomarkers such as different micro-RNAs [11], have been related to AAA progression. Among geometric features, thrombus presence [12] and different AAA curvature metrics [13,14,15] have been positively associated with growth rate. However, the role of other mechanisms, such as inflammation and disturbed hemodynamics, on AAA growth is still debated.

Inflammatory and proteolytic activity in the aortic wall, as measured by 18F-fluorodeoxyglucose ([18F]FDG) Positron Emission Tomography (PET) uptake [16,17], has been correlated with AAA growth rates, but with remarkably inconsistent findings. While specific studies have reported a positive association between [18F]FDG uptake and AAA growth [18,19], others have reported a negative association [20,21] or the absence of an association [17,22] based on the degree of aortic wall degeneration.

Studies investigating the association between AAA hemodynamics using four-dimensional (4D) Flow cardiovascular magnetic resonance (CMR) and AAA growth are scarce. While AAAs are known to present abnormal vortical flow patterns [23,24], low velocity and wall shear stress (WSS) [23,25,26] and high regurgitant flow [26], the only study [24] that investigated AAA growth found no significant correlation between in vivo velocity and WSS and growth rate. Conversely, in-silico methods like computational fluid dynamics simulations have suggested that low WSS may be associated with AAA growth [27,28,29]. However, in vivo validation of these findings is needed, as these simulations require simplifications and assumptions.

This study aimed to investigate the possible predictive value of local, in vivo glucose metabolism and flow conditions for abdominal aneurysm growth.

## 2. Materials and Methods

### 2.1. Study Population

The study adhered to the Declaration of Helsinki and was approved by the Institutional Ethics Committee of Vall d’Hebron University Hospital (PR(AG)253/2019 on 28 June 2019).

Adult (age > 18 years) patients with infrarenal fusiform AAA with a maximum AAA diameter ≥ 40 mm and life expectancy > 3 years were consecutively included in this prospective observational study between 2019 and 2023 at Vall d’Hebron University Hospital. Life expectancy longer than three years was clinically estimated by the treating vascular surgeon, based on functional status, major comorbidities, active malignancy and advanced organ dysfunction [5,30]. No formal survival prediction score was used. Exclusion criteria were pseudoaneurysms, ruptured or symptomatic aneurysms, aortic surgery or a history of acute aortic syndromes, severe aortic valve disease, bicuspid aortic valve, connective tissue disorder or contraindications for CMR or PET. All patients were invited for a baseline hybrid [18F]FDG-PET/MR scan.

In addition, as part of clinical follow-up, computed tomography (CT) and/or ultrasound studies (US) were performed to measure the maximum AAA diameter. A prospective approach, using the first and last diameter measurements obtained after the baseline PET/CMR, was preferred to measure the AAA growth rate. However, for patients that met surgery criteria, the closest pair of diameters measured before surgery was considered. The same imaging modality was used to measure the diameters for growth rate evaluation. After a single observer reviewed baseline and follow-up maximum diameters, growth rates were computed as the difference in the maximum AAA diameter between follow-up and baseline studies divided by the follow-up duration in years, providing annual growth rates (in millimeters per year). CT was preferred over the US when both were available.

### 2.2. Image Acquisition

**Figure 1:**
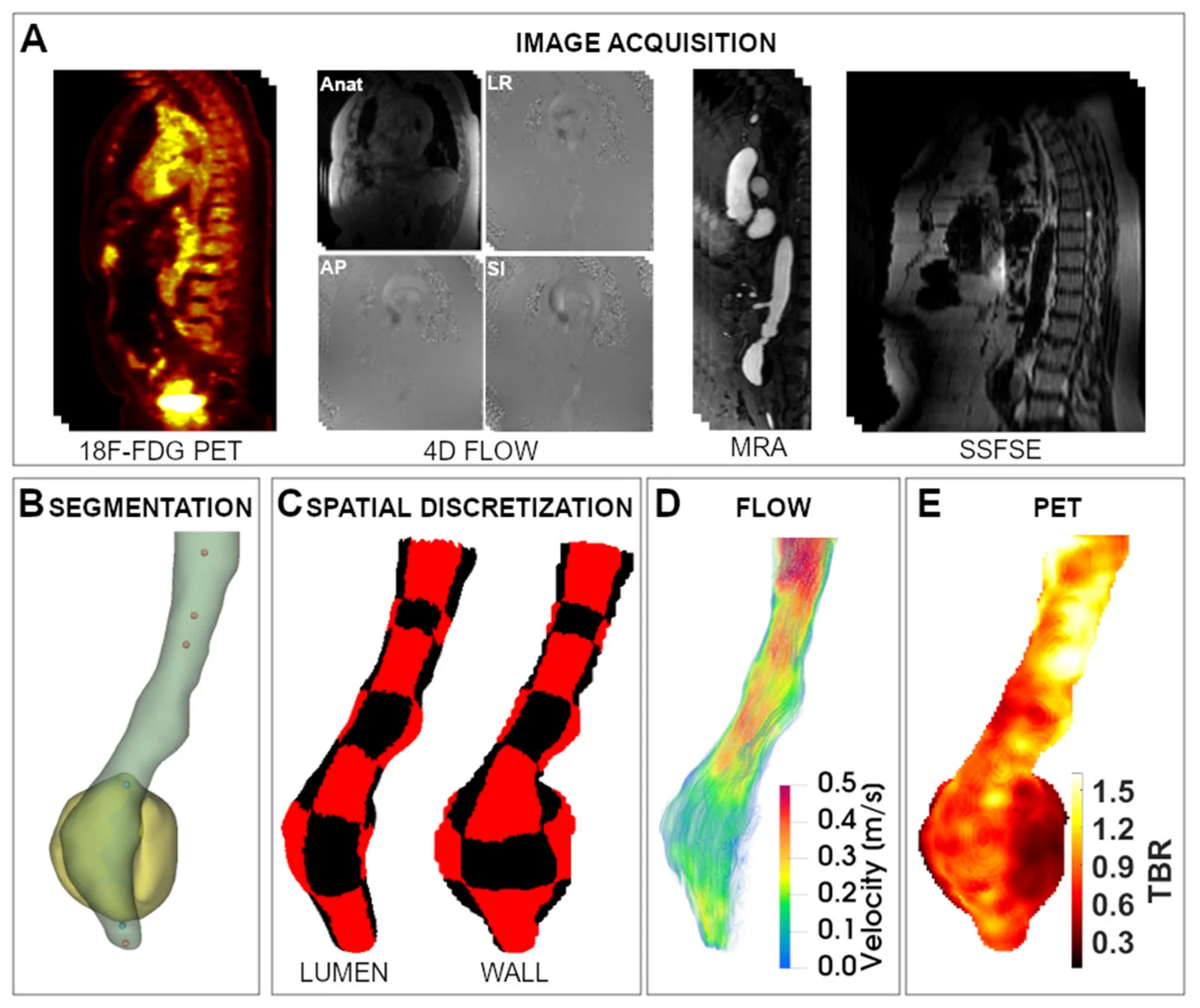
Flowchart of the methodology followed to assess the different PET/CMR-derived parameters included in the AAA growth analysis. (**A**) PET/CMR acquisition; (**B**) Lumen and thrombus segmentation and landmarks annotation; (**C**) Spatial discretization in standardized aortic patches; (**D**) Quantification of flow characteristics by 4D flow CMR; (**E**) TBR quantification on PET images. 18F-FDG PET: 18F-fluorodeoxyglucose positron emission tomography; Anat: Anatomical; LR: Left–Right; AP: Anterior–Posterior; SI: Superior–Inferior; MRA: Magnetic resonance angiography; SSFSE: Single-shot fast spin echo; TBR: Target-to-background ratio.

Image acquisitions were performed in a hybrid whole-body PET-MRI 3T scanner (SIGNATM PET/MRI, GE, General Electric Healthcare, Waukesha, WI, USA). The CMR scan protocol included (i) a 4D flow of the thoracic-abdominal aorta using a 3D spoiled gradient-echo sequence with retrospective cardiac gating during free breathing, velocity encoding set to 125% of the maximum aortic valve velocity from 2D cine phase contrast CMR, a voxel size of 1.72 × 1.72 × 2 mm and a temporal resolution of 22.6 ms, (ii) contrast-enhanced magnetic resonance angiography (MRA) with the administration of gadolinium chelated contrast, with a voxel size of 0.86 × 0.86 × 3.4 mm, and (iii) a blood-suppressed single-shot fast-spin echo sequence, with a voxel size of 0.70 × 0.70 × 5 mm (Figure 1A). Aliasing was mitigated by applying the Laplacian-based algorithm for phase unwrapping, as described [31].

Patients were administered 4 MBq/kg of [18F]FDG intravenously one hour before the image acquisition for PET imaging. Prior to imaging, patients were instructed to fast for 6 h and follow a low-carbohydrate diet for 48 h. Fast spoiled gradient echo MR sequences were used to perform the attenuation correction. For this purpose, four distinct contrast maps were created to acquire only water and fat, in-phase and out-phase. Image reconstruction was performed through the time-of-flight algorithm (Q.Clear, GE, General Electric Healthcare, Waukesha, WI, USA), which reduces noise (square pixel size of 3.13 [3.13, 3.13] mm).

### 2.3. Image Analysis

The abdominal aorta and the thrombus were segmented on MRA (Figure 1B) in 3D Slicer [32], utilizing the black-blood sequence to provide additional guidance for thrombus segmentation. Using these segmentations, aneurysm and thrombus volumes and the area of the aortic wall covered by the thrombus were computed from the tetrahedral mesh elements. In patients with thrombus, the intraluminal thrombus volume indexed to the total aneurysm volume (ILT ratio) was computed. In patients without thrombus, thrombus volume and area were set to zero. The segmentations were merged, generating a unique 3D mesh of the aortic wall. The aortic centerlines were computed both for the aorta and the aortic lumen (excluding thrombus), and four anatomical reference points were located at the diaphragmatic level, superior mesenteric artery, renal arteries and aortoiliac bifurcation. Two additional reference points corresponding to the beginning and end of the abdominal aneurysm were identified. The subsequent image analysis was performed in MATLAB (version R2023b; MathWorks, Inc., Natick, MA, USA). First, using the anatomical points and the corresponding centerline, the abdominal aortic wall and the lumen were discretized into 28 standardized patches (Figure 1C). To this aim, the abdominal aorta was longitudinally divided into one region between the diaphragm and the superior mesenteric artery, one region between the mesenteric and the renal arteries and five equally spaced areas in the infrarenal aorta. Finally, each longitudinal region was divided into four circumferential regions.

#### 2.3.1. Hemodynamic Parameters Quantification

Aortic flow parameters were quantified based on a finite element method, following a previously described methodology (Figure 1D) [33,34]. A tetrahedral finite element mesh was generated based on the 4D Flow CMR images and the lumen segmentation. Then, blood velocity maps were calculated by interpolating velocities from 4D Flow CMR images to each mesh node.

Subsequently, the WSS magnitude and its axial and circumferential components were obtained. The time-averaged wall shear stress (TAWSS) over the cardiac cycle was calculated. Then, the relative residence time was quantified to evaluate the duration during which blood resides close to the wall [25]. In addition, the oscillatory shear index (OSI) was computed to quantify the directional changes of WSS during a cardiac cycle.

The axial angle, defined as the angle between the velocity vector and the axial direction, was measured to evaluate flow alignment. The rotational flow was assessed using the vorticity, helicity density, axial circulation and systolic flow reversal ratio (SFRR). Vorticity was computed to measure the local spinning motion of the blood flow. Helicity density was then assessed to quantify the intensity and direction of helical flow patterns. Axial circulation was calculated to evaluate the rotational flow within the planes, while the SFRR quantified the through-plane rotational flow during the systole.

The median values of velocity, WSS, axial angle, vorticity, helicity density and axial circulation in each standardized patch were extracted at peak systole, which was determined through the net flow curve at the diaphragm level. Furthermore, for patient-level characterization, the median of each variable was computed across all patches comprising the AAA.

#### 2.3.2. [18F]FDG Parameter Quantification

Before extracting PET data, the colocalization between the AAA wall segmentation and PET images was visually evaluated, and manual correction of misalignments was performed by rigid body transformation, if needed. To calculate the standardized uptake value (SUV) in the aortic wall, the aortic wall mesh was expanded 6 mm (3 mm inward and 3 mm outward), as previously described and validated [35]. Then, the resulting volume was voxelized with a uniform spatial resolution of 1 mm, and SUV values were interpolated. SUV was computed as the ratio of the decay-corrected FDG concentration by the injected dose and body weight [36].

For blood uptake correction, the target-to-background (TBR) (Figure 1E) [37] was computed as the ratio of SUV in the target area to the mean blood uptake in a sphere of 16 mm of diameter localized in the left atrium [38]. The median of the TBR was calculated at each abdominal standardized patch. For patient-level characterization, the median across all patches comprising the AAA was obtained.

### 2.4. Statistical Analysis

Variables are reported as medians and non-adjusted interquartile ranges (IQRs), as their distribution is not normal. The Spearman’s rank correlation coefficient (r_s_) was used to assess the strength and direction of the associations between possible independent variables and the aneurysm diameter and growth rate in bivariate linear models. Differences between groups for continuous parameters were assessed with the Mann–Whitney U test. A multivariable linear regression model was developed to identify variables independently associated with AAA growth rate. Variables were considered for inclusion in the multivariable model if they had a *p*-value < 0.15 in either the bivariate or the diameter-adjusted analysis. When multiple variables described the same domain (clinical, morphological, flow), preference was given to variables with *p*-values < 0.15 in both bivariate and diameter-adjusted analyses. Final variable selection was performed using a forward stepwise feature selection method, with model coefficients and *p*-values recalculated at each step. Variables were added to the model until no remaining candidate variables had a *p*-value < 0.1. Multicollinearity between variables included in the multivariable model was checked using the variance inflation factors. Data imputation was used to handle missing data in PET and flow variables by replacing missing values with median values across all patients. Statistical computations were performed in Rstudio (version 2024.04.2 + 764, RStudio, Boston, MA, USA) in R (version 4.3.2, R Foundation for Statistical Computing, Vienna, Austria).

## 3. Results

### 3.1. Patient Population

A total of 32 patients were included in the study. The median age was 73 [68; 77] years, and 84.4% were men. Very prevalent comorbidities were hypertension (84.4%) and dyslipidemia (75.0%), while approximately a third of the patients had diabetes mellitus or peripheral artery disease. The median maximum AAA diameter at the time of PET/CMR was 48.1 [44.8; 55.0] mm, and 24 (75.0%) of the AAA presented thrombus. After a median follow-up time of 2 [1.13; 3.47] years, the AAA growth rate was 2.7 [1.3; 4.0] mm/year, while none of the aneurysms ruptured or became symptomatic. Growth rate was measured by CT in 19 patients and by US in 13 patients. Nineteen patients underwent surgical repair of the AAA during the study period. Demographic and clinical characteristics are detailed in Table 1.

One patient had missing [18F]FDG PET data, and five patients had missing 4D Flow CMR data because images could not be acquired. The growth rate was available in all 32 patients.

### 3.2. Associations with Maximum AAA Diameter and Growth

**Figure 2:**
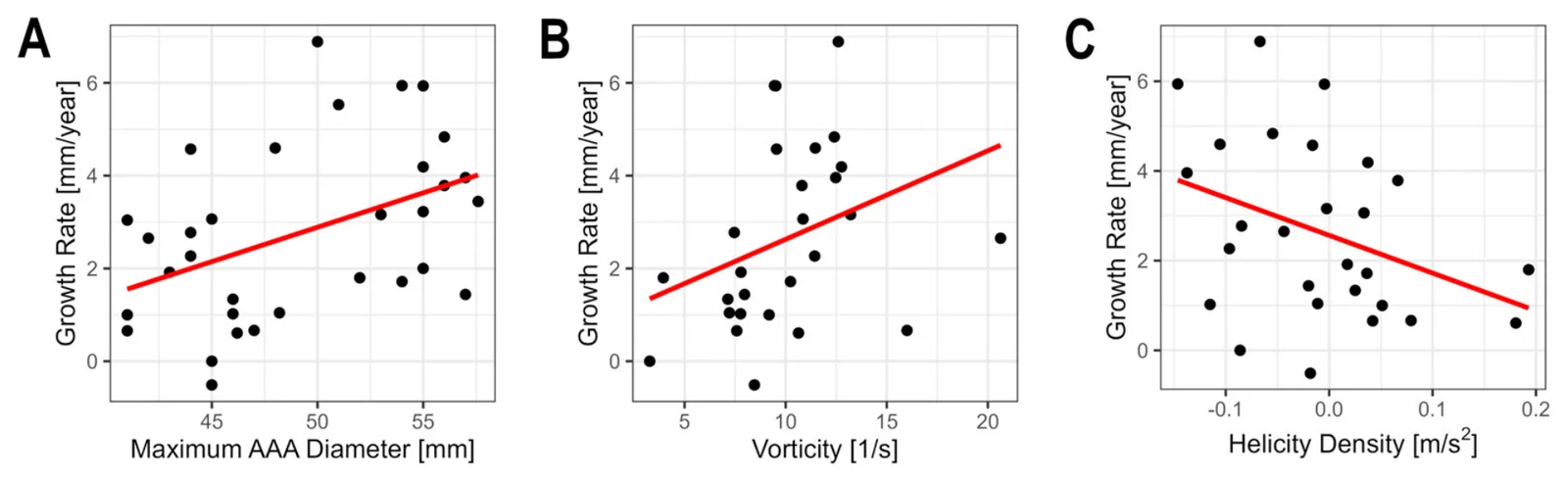
Patient-level associations between diameter and flow variables and growth rate. (**A**) Maximum aneurysm Diameter, (**B**) Vorticity and (**C**) Helicity Density. Panels (**B**,**C**) include only those patients for whom 4D-flow CMR data were available.

Bivariate analyses were performed to identify variables correlated with aneurysm diameter and growth rate (Table 2, Supplementary Table S1). Neither demographic nor clinical variables were correlated with diameter or growth rate (Table S1). Maximum AAA diameter was significantly correlated with growth rate (r_s_ = 0.443, *p* = 0.011) (Figure 2A), whereas AAA volume and length were not. Correlations were therefore adjusted for maximum AAA diameter.

**Figure 3:**
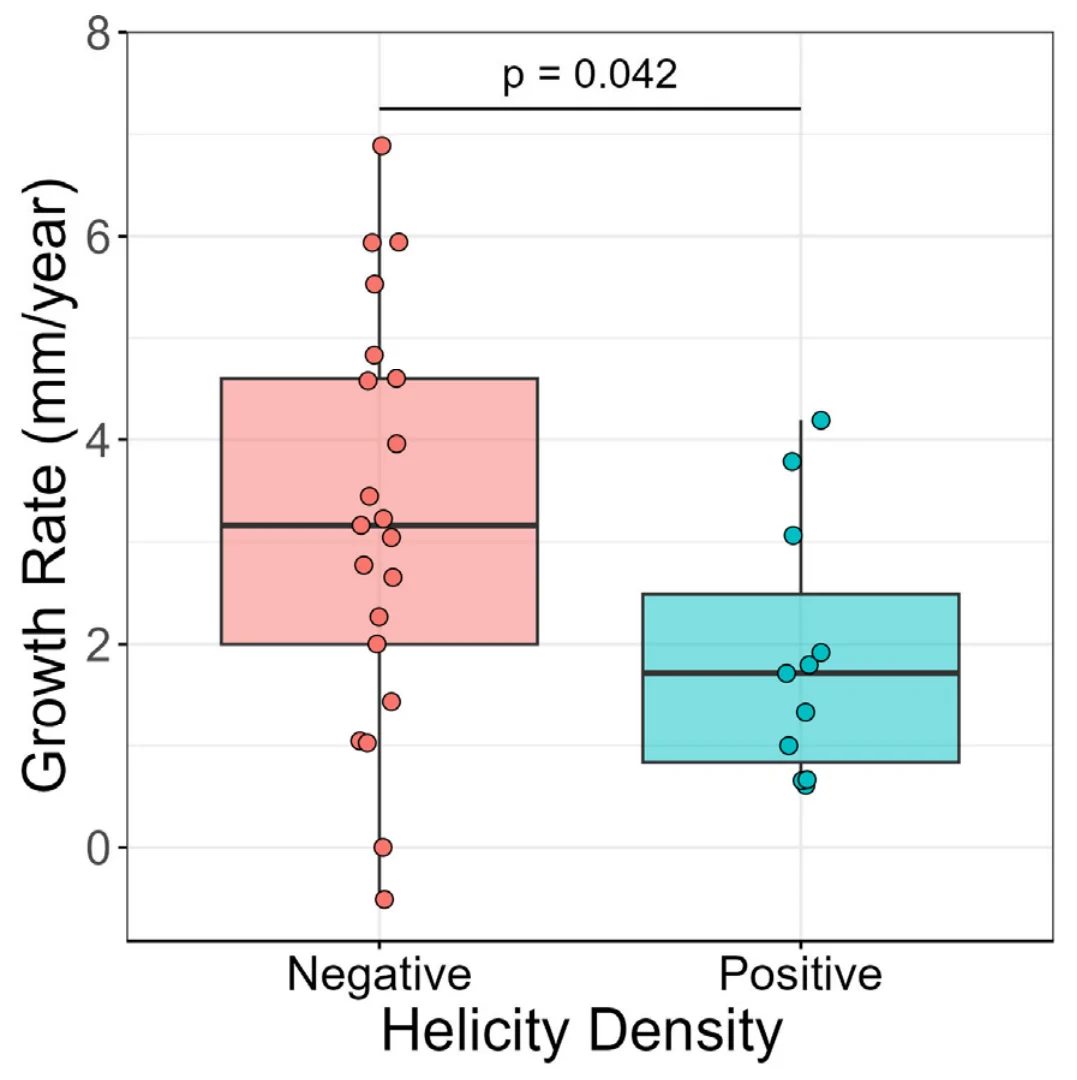
Growth rate and helicity density. Boxplot of AAA growth rate in AAA patients, depending on positive or negative helicity density in the aneurysm. Each data point represents an individual patient in the corresponding boxplot.

Among the hemodynamic variables, vorticity showed a significantly positive association with growth rate (r_s_ = 0.453, *p* = 0.009) (Figure 2B). However, when adjusting for maximum AAA diameter, vorticity was not significantly correlated with growth rate (*p* = 0.077), suggesting that the bivariate association partially reflected the impact of diameter. While not statistically significant, helicity density showed a tendency for negative association with AAA growth rate (*p* = 0.079) (Figure 2C). Once corrected for maximum AAA diameter, a statistically significant (*p* = 0.045) association was evident. Notably, aneurysms with negative helicity density (counterclockwise rotation) grew significantly faster (*p* = 0.042) than those with positive helicity (Figure 3). No other flow descriptor significantly correlated with aneurysm diameter or growth.

Concerning PET data, although not statistically significant, TBR tended to be negatively associated with AAA diameter (r_s_ = −0.268, *p* = 0.138) and growth rate (r_s_ = −0.293, *p* = 0.101). However, TBR was not correlated with growth rate after correction for maximum AAA diameter.

AAA thrombus area and volume and ILT ratio were moderately correlated with maximum AAA diameter (r_s_ = 0.518, *p* = 0.002; r_s_ = 0.630, *p* < 0.001 and r_s_ = 0.498, *p* = 004, respectively) but not with aneurysm growth (*p* = 0.888, *p* = 0.608 and *p* = 0.704, respectively). However, when corrected by aneurysm diameter, thrombus volume demonstrated a negative independent correlation with aneurysm growth (*p* = 0.042).

### 3.3. Multivariable Analysis

**Figure 4:**
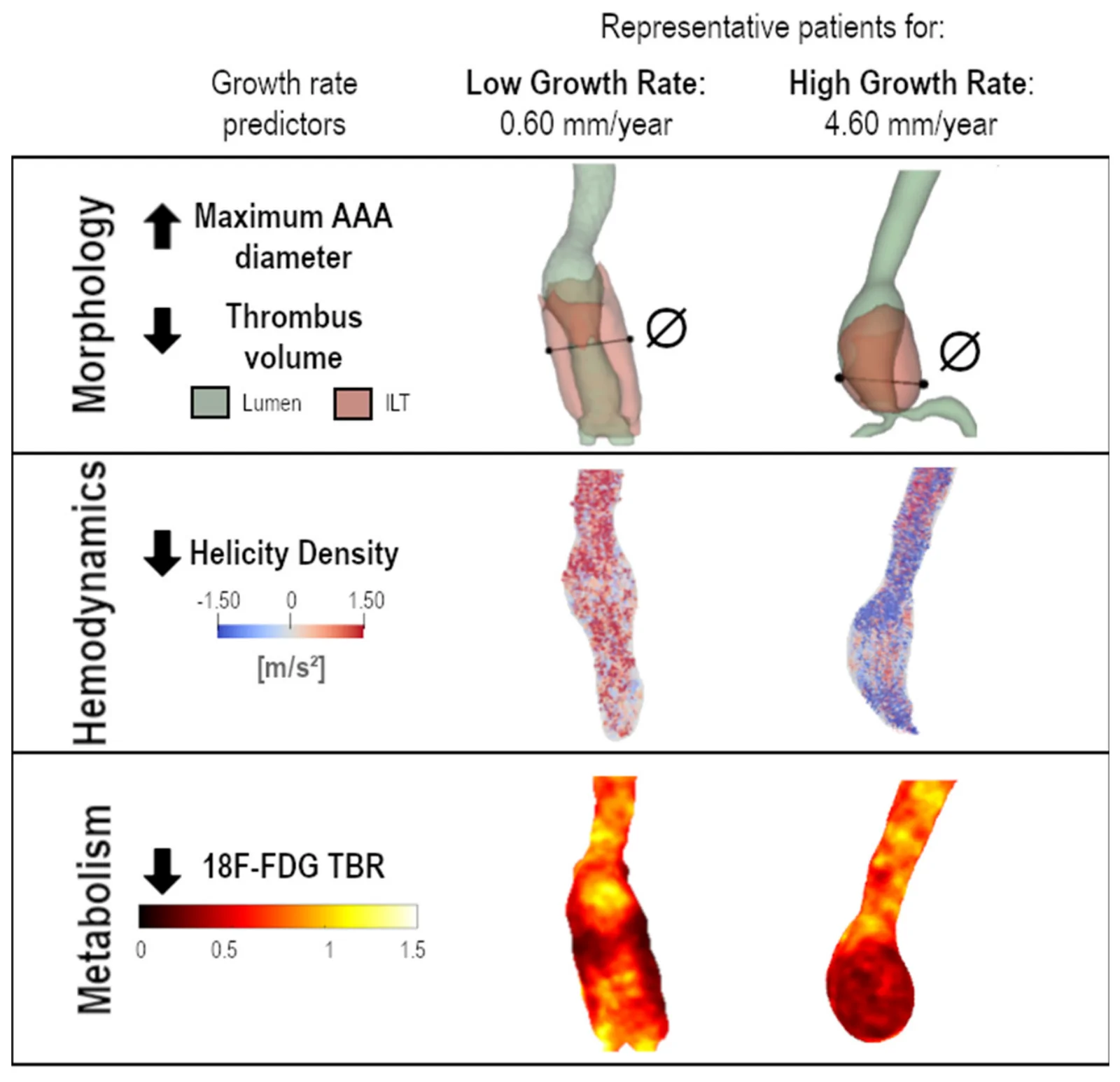
Summary of growth rate predictors and representative patients with low and high growth rates. Patients with higher growth rates exhibited larger maximum abdominal aortic aneurysm (AAA) diameters, lower thrombus volumes, lower helicity densities and lower 18F-Fluorodeoxyglucose (FDG) target-to-background (TBR) values than those with lower growth rates. ↑/↓ arrows denote higher/lower values, respectively.

In the multivariable analysis, the variables entered into the model were vorticity and helicity density for flow dynamics, TBR for metabolism, maximum AAA diameter and thrombus volume for AAA morphology and diabetes mellitus for clinical descriptors. In the final multivariable model, maximum AAA diameter, thrombus volume, helicity density and TBR were independently associated with growth rate (Table 3 and Figure 4). A larger aneurysm diameter, stronger counterclockwise helicity and lower TBR were identified to be related to growth rate, whereas thrombus volume showed an inverse association.

## 4. Discussion

This study evaluated the potential predictive value of blood flow dynamics and [18F]FDG uptake for AAA growth using PET/CMR data. In the multivariable analysis, maximum AAA diameter, thrombus volume, helicity density and [18F]FDG uptake showed an independent association with aneurysm growth.

As expected, the diameter of the aneurysm was positively correlated with the growth rate. This correlation has been observed in previous studies [39,40,41,42], and it is reflected in clinical guidelines [43]. While the fundamental role of the aneurysm diameter in the progression of AAA is well established, the present results shed light on the relationship between such a measure of AAA severity and other factors possibly associated with AAA progression, such as hemodynamics, glucose consumption and intra-luminal thrombus.

Regarding hemodynamics, rotational flow descriptors were associated with growth rate. Previous 4D flow CMR studies had qualitatively evaluated vortical flow in AAA by visually assessing the presence and degree of vortices [23,24]. In line with Aalbregt et al. [24], our results showed no correlation between vorticity and maximum diameter. In contrast to that study, quantitatively assessed vorticity was found to be significantly associated with growth rate in bivariate analysis, although this significance was lost in multivariable analysis. Differently, helicity density revealed an independent negative association with growth rate in multivariable analysis. Moreover, patients with negative helicity density exhibited a significantly faster aneurysm growth (*p* = 0.042), suggesting that counterclockwise rotational flow may promote aneurysm growth. Previous in vitro studies revealed the impact of flow direction relative to endothelial cell alignment on the activation of atheroprotective and pro-inflammatory pathways that play significant roles in determining endothelial function and disease [44,45].

No clear evidence has been presented on the normal helical pattern direction in the abdominal aorta in vivo nor its association with disease. Conversely, studies reported positive helical flow in the thoracic aorta of healthy volunteers [46,47,48] and associated negative helical flow in the thoracic descending aorta with local dilation and interventions [47,49]. Therefore, normal values for abdominal aorta helicity are needed, while the clinical significance of counterclockwise helicity in AAA requires validation in larger cohorts.

Concerning PET assessment, the present results show that [18F]FDG absorption was negatively correlated with growth rate in multivariable analysis. Low TBR may indicate structural wall weakness, possibly related to a decellularization process caused by hypoxic conditions, which can lead to aneurysm progression. This aligns well with AAA pathogenesis, which is characterized by the degradation of elastin within the extracellular matrix [50], which, together with the proteolytic and oxidative activity [51,52], further weakens the aneurysm wall and reduces glucose consumption [53].

Finally, thrombus volume was inversely correlated with growth rate in bivariate analysis corrected for maximum AAA diameter. This association may reflect a protective role of thrombus against aneurysm growth, potentially related to a reduction in wall stress, although our data do not allow us to establish a mechanistic basis for this finding. Previous studies have proposed that intraluminal thrombus may function as a mechanical buffer, attenuating hemodynamic stress transmitted from the arterial lumen to the vessel wall [54,55,56,57], while others suggested that thrombus may instead be detrimental by impairing the structural integrity of the aortic wall [58,59]. Given these conflicting hypotheses, further studies are needed to clarify the role of intraluminal thrombus in AAA progression.

From a clinical perspective, integrated PET/MRI holds considerable promise for the comprehensive assessment of AAA by combining morphological, hemodynamic and molecular imaging within a single examination. This multiparametric approach has the potential to overcome the limitations of the maximum AAA diameter, which remains the primary criterion for surveillance and intervention despite its recognized inability to fully capture individual rupture risk [5]. However, several barriers currently hinder its routine clinical implementation, including the limited availability and high cost of integrated PET/MRI systems, prolonged acquisition times, the lack of standardized acquisition and post-processing protocols and the absence of large, prospective, adequately powered studies validating these imaging biomarkers against clinically relevant endpoints such as aneurysm growth and rupture across diverse patient populations, including underrepresented groups such as women and younger patients. Large multicenter longitudinal studies are therefore required to determine whether morphological, hemodynamic and metabolic imaging biomarkers can provide robust, reproducible and enhanced risk stratification of patients with AAA.

There were several limitations in our study. This is a single-center study involving a modest number of patients: thus, the generalizability of these findings requires further evaluation, and the results of the multivariable model should be considered exploratory. As the cohort was consecutively enrolled and reflects the typical AAA population, which is predominantly composed of older men, the generalizability of the findings to underrepresented groups, such as women and younger patients, remains uncertain. Additionally, the discriminative performance of the evaluated descriptors for clinically relevant growth-rate subgroups (low vs high growth) could not be assessed in the present cohort and should be addressed in future studies. This study calculated a single growth rate per AAA from the maximum diameter, assuming linear growth in time and using different imaging techniques based on clinical practice availability, which may induce bias. Of note, it has been found that about 70–90% of AAA patients exhibit linear growth [60,61]. The potential local correlation between growth and other markers (such as flow and PET uptake) could not be assessed since AAA growth was evaluated using standard clinical practice. Local growth rate assessment [62] should be considered in further studies, including multiple centers and more patients.

## 5. Conclusions

Maximum AAA diameter was positively related to AAA growth, while thrombus volume, helicity density and [18F]FDG PET uptake demonstrated a negative association with growth. If confirmed by other studies, these findings support that evaluating rotational flow, inflammation and thrombus size, in addition to maximum diameter, may be valuable for predicting AAA progression.

## Figures and Tables

**Table 1:** Demographic and clinical characteristics of patients.

Demographic and Clinical Variables	
Number of patients, N	32
Age [years]	73 [68; 77]
Male sex, N [%]	27 (84.4%)
BSA [m^2^]	1.9 [1.8; 2]
SBP [mmHg]	154 [137; 174]
DBP [mmHg]	85.0 [75.5; 94.0]
LDL [mg/dL]	96.0 [76.8; 137.0]
HDL [mg/dL]	47.5 [41.7; 56.2]
Cholesterol [mg/dL]	176.0 [147.0; 221.5]
Glycated hemoglobin [%]	5.8 [5.6; 6.5]
eGFR [mL/min/1.73 m^2^]	66.0 [58.5; 80.8]
Smoking status, N [%]	
Current smoker	11 (34.4%)
No smoker	17 (53.1%)
Past smoker	3 (9.4%)
Comorbidities, N [%]	
Chronic kidney disease	5 (15.6%)
Hypertension	27 (84.4%)
Chronic obstructive pulmonary disease	3 (9.4%)
Diabetes mellitus	10 (31.2%)
Dyslipidemia	24 (75.0%)
Coronary artery disease	5 (15.6%)
Peripheral artery disease	11 (34.4%)
Pharmacological treatment, N [%]	
Antiplatelet therapy	21 (65.6%)
Lipid-lowering therapy	19 (59.4%)
Aneurysm measurements	
Presence of thrombus, N [%]	24 (75.0%)
Maximum aneurysm diameter [mm]	48.1 [44.8; 55.0]
Lumen diameter [mm]	36.6 [32.0; 41.2]
Aneurysm length [mm]	86.0 [71.4; 103.0]
Growth rate [mm/year]	2.7 [1.3; 4.0]
ILT ratio [%]	52 [25; 63]

Values are the median [first; third quartile] or N (%). BSA = body surface area; SBP and DBP: systolic and diastolic blood pressure, respectively; LDL: low-density lipoprotein; HDL: high-density lipoprotein; eGFR: estimated glomerular filtration rate; ILT: intraluminal thrombus.

**Table 2:** Bivariate correlations between imaging biomarkers, aneurysm diameter and growth rate.

	Values	Bivariate Analysis				Adjusting for AAA Diameter	
		Aneurysm Diameter		Growth Rate		Growth Rate	
		r_s_	*p*-Value	r_s_	*p*-Value	β	*p*-Value
Hemodynamics							
Velocity [m/s]	0.103 [0.090; 0.141]	0.185	0.312	0.137	0.454	4.199	0.620
Axial angle [º]	15.243 [12.291; 26.258]	0.050	0.786	0.116	0.526	0.002	0.949
SFRR [%]	16.371 [8.276; 33.180]	0.097	0.599	0.035	0.850	−0.002	0.923
RRT	14.850 [13.090; 22.110]	−0.080	0.665	−0.111	0.544	−0.049	0.330
TAWSS [Pa]	0.094 [0.076; 0.121]	0.183	0.317	0.272	0.132	6.006	0.635
Magnitude WSS [Pa]	0.185 [0.121; 0.238]	0.218	0.231	0.203	0.264	1.083	0.781
Axial WSS [Pa]	0.177 [0.106; 0.227]	0.233	0.199	0.226	0.214	1.571	0.672
Circumferential WSS [Pa]	0.034 [0.026; 0.039]	0.246	0.175	0.338	0.059	18.712	0.536
OSI [-]	0.149 [0.111; 0.191]	0.173	0.343	0.091	0.621	2.048	0.685
Vorticity [1/s]	9.550 [7.780; 11.930]	0.239	0.188	0.453	0.009	0.170	0.077
Helicity Density [m/s^2^]	−0.011 [−0.076; 0.037]	−0.046	0.802	−0.316	0.079	−7.915	0.045
Axial Circulation [cm^2^/s]	−0.427 [−3.324; 2.880]	0.118	0.521	−0.150	0.414	−0.043	0.276
18F-FDG PET							
TBR [-]	0.800 [0.607; 1.006]	−0.268	0.138	−0.293	0.104	−1.342	0.259
AAA morphology							
AAA diameter [mm]	48.100 [44.750; 55.000]	N/A	N/A	0.443	0.011	N/A	N/A
AAA length [mm]	91.522 [67.664; 104.986]	0.307	0.087	−0.183	0.316	−0.030	0.021
AAA volume [cm^3^]	114.313 [78.679; 141.454]	0.680	0.000	0.147	0.421	−0.018	0.012
Thrombus Area [cm^2^]	88.726 [32.288; 114.850]	0.518	0.002	0.026	0.888	−0.011	0.100
Thrombus Volume [cm^3^]	51.908 [21.623; 85.053]	0.630	<0.001	0.094	0.608	−0.015	0.042
ILT ratio [%]	52.037 [25.252; 62.600]	0.498	0.004	0.070	0.704	0.008	0.665

Values are the median [first; third quartile]. AAA = Abdominal Aortic Aneurysm; SFRR = Systolic Flow Reversal Ratio; RRT = Relative Residence Time; TAWSS = time average wall shear stress; WSS = Wall Shear stress; OSI = Oscillatory Shear Index; TBR = Target-to-Background Ratio; 18F-FDG PET = 18F-Fluorodesoxyglucose Positron Emission Tomography.

**Table 3:** Multivariable linear analysis for abdominal aortic aneurysm growth rate.

	Unstandardized Coefficients	Standardized Coefficients	*p*-Value
Aneurysm diameter [mm]	0.215	0.634	0.001
Thrombus volume [cm^3^]	−0.017	−0.482	0.011
Helicity density [m/s^2^]	−9.687	−0.400	0.008
TBR [-]	−2.074	−0.303	0.046

Unstandardized coefficients are defined as millimeters per year of growth per variable unit change. TBR = Target-to-Background (TBR).

## Data Availability

The data that support the findings of this study are available from the corresponding author upon reasonable request.

## Supplementary Materials

The following supporting information can be downloaded at: https://www.mdpi.com/article/10.3390/life16081308/s1, Figure S1: Patient-level associations between maximum abdominal aortic aneurysm (AAA) diameter and its growth and thrombus descriptors. Maximum AAA diameter (A,C) and growth rate (B,D) versus thrombus area (A,B) and volume (C,D), respectively; Table S1: Bivariate correlations between clinical and demographical variables, aneurysm diameter, and growth rate.

## Abbreviations

The following abbreviations are used in this manuscript:

4D	Four-dimensional
AAA	Abdominal aortic aneurysm
CMR	Cardiovascular magnetic resonance
CT	Computed tomography
FDG	Fluorodeoxyglucose
IQR	Interquartile range
MRA	Magnetic resonance angiography
OSI	Oscillatory shear index
PET	Positron Emission Tomography
SFRR	Systolic flow reversal ratio
SUV	Standardized uptake value
TAWSS	Time-averaged WSS
TBR	Target-to-background
US	Ultrasound
WSS	Wall shear stress
