# Supplementary material for: Predicting Abdominal Aortic Aneurysm Progression: The Role of 18F-Fluorodeoxyglucose Aortic Wall Uptake and Flow Dynamics
Source: Life (Basel). 2026 Aug 10;16(8):1308. doi: 10.3390/life16081308 (PMC13514937; doi:10.3390/life16081308)
Supplement: Supplementary file 1 [file life-16-01308-s001.zip › life-4467500-supplementary.pdf]

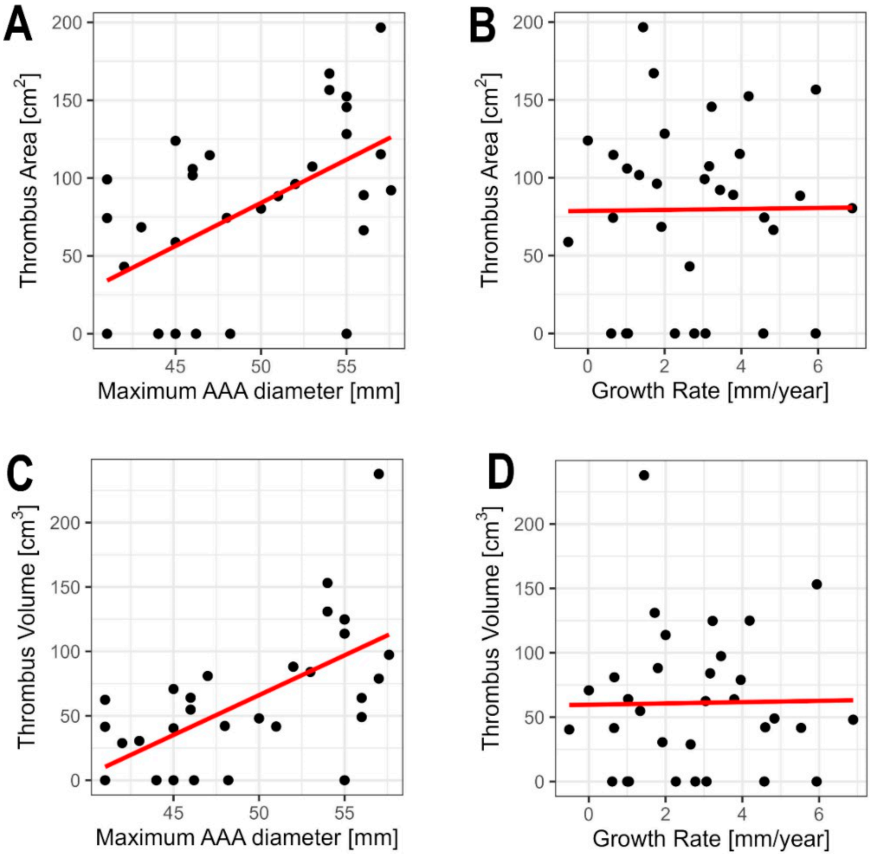

Figure S1. Patient-level associations between maximum abdominal aortic aneurysm (AAA) diameter and its growth and thrombus descriptors. Maximum AAA diameter (A, C) and growth rate (B, D) versus thrombus area (A,B) and volume (C,D), respectively.

Table S1. Bivariate correlations between clinical and demographical variables, aneurysm diameter, and growth rate.

	Values	Bivariate analysis				Adjusting for AAA diameter	
		Aneurysm Diameter		Growth Rate		Growth Rate	
		r_s	p-value	r_s	p-value	β	p-value
**Clinical / demographical**							
Age [years]	73 [68; 77]	0.009	0.961	-0.081	0.660	-0.010	0.820
Sex [male N(%)]	5 (12.8%)	NA	0.090	NA	0.150	1.125	0.432
BSA [m^2]	1.9437 [1.756; 2.0328]	0.089	0.629	0.172	0.347	-0.487	0.593
Hypertension [N (%)]	27 (69.2%)	NA	0.942	NA	0.172	-1.177	0.206
Obesity [N (%)]	2 (5.1%)	NA	0.512	NA	0.520	-1.464	0.253
Peripheral artery disease [N (%)]	11 (28.2%)	NA	0.647	NA	0.476	0.349	0.598
Coronary artery disease [N (%)]	5 (11.9%)	NA	1.000	NA	0.359	-0.875	0.304
Current smoker [N (%)]	28 (71.8%)	NA	0.011	NA	0.385	-0.053	0.921
Diabetes mellitus [N (%)]	10 (25.6%)	NA	0.795	NA	0.079	-1.232	0.060

Values are median [first; third quartile]. AAA = abdominal aortic aneurysm; BSA = body surface area
